# Evaluating the potential of non‐immunosuppressive cyclosporin analogs for targeting *Toxoplasma gondii* cyclophilin: Insights from structural studies

**DOI:** 10.1002/pro.5157

**Published:** 2024-09-23

**Authors:** Filippo Favretto, Eva Jiménez‐Faraco, Gianluca Catucci, Adele Di Matteo, Carlo Travaglini‐Allocatelli, Sheila J. Sadeghi, Paola Dominici, Juan A. Hermoso, Alessandra Astegno

**Affiliations:** ^1^ Department of Biotechnology University of Verona Verona Italy; ^2^ Department of Crystallography and Structural Biology Institute of Physical Chemistry Blas Cabrera (IQF), CSIC Madrid Spain; ^3^ Department of Life Sciences and Systems Biology University of Turin Turin Italy; ^4^ CNR Institute of Molecular Biology and Pathology Rome Italy; ^5^ Department of Biochemical Sciences Sapienza University of Rome Rome Italy

**Keywords:** cyclophilin inhibitors, cyclophilins, cyclosporin A, peptidyl‐prolyl isomerases, *Toxoplasma gondii*, X‐ray crystal structure

## Abstract

Toxoplasmosis persists as a prevalent disease, facing challenges from parasite resistance and treatment side effects. Consequently, identifying new drugs by exploring novel protein targets is essential for effective intervention. Cyclosporin A (CsA) possesses antiparasitic activity against *Toxoplasma gondii*, with cyclophilins identified as possible targets. However, CsA immunosuppressive nature hinders its use as an antitoxoplasmosis agent. Here, we evaluate the potential of three CsA derivatives devoid of immunosuppressive activity, namely, NIM811, Alisporivir, and dihydrocyclosporin A to target a previously characterized cyclophilin from *Toxoplasma gondii* (TgCyp23). We determined the X‐ray crystal structures of TgCyp23 in complex with the three analogs and elucidated their binding and inhibitory properties. The high resolution of the structures revealed the precise positioning of ligands within the TgCyp23 binding site and the details of protein–ligand interactions. A comparison with the established ternary structure involving calcineurin indicates that substitutions at position 4 in CsA derivatives prevent calcineurin binding. This finding provides a molecular explanation for why CsA analogs can target Toxoplasma cyclophilins without compromising the human immune response.

## 1 INTRODUCTION

Toxoplasmosis, caused by the parasite *Toxoplasma gondii*, is a widespread disease with diverse manifestations, including acute and chronic symptoms, and potential complications such as ocular, congenital, or neuro‐toxoplasmosis (Halonen & Weiss, 2013; Skariah et al., 2010). The disease poses significant health risks, especially to fetuses, newborns, and individuals with weakened immune systems (McAuley, 2014). Recent studies have also identified associations between toxoplasmosis and various neuropathies and cancer types (Ngô et al., 2017).

Despite its prevalence, toxoplasmosis remains a neglected human and veterinary health concern. Current treatments involving antiparasitic and antibacterial drugs face challenges such as intolerance, side effects, and the development of parasite resistance. Moreover, these drugs primarily target acute toxoplasmosis, with limited impact on the chronic form of the disease (Alday & Doggett, 2017; Hajj et al., 2021; Montazeri et al., 2017). Identifying new drugs by exploring novel host–pathogen interaction pathways, drug resistance mechanisms, and new molecular drug targets is therefore crucial for innovative treatment approaches, considering the drawbacks of existing drugs.

Cyclophilins (Cyps) represent a potential class of drug targets for various diseases, belonging to the immunophilins alongside FK506‐binding proteins (Nigro et al., 2013; Zhou et al., 2012). These ubiquitous cellular proteins, aside from acting as chaperones, possess peptidyl‐prolyl isomerase (PPIase) activity, aiding in protein folding or refolding by accelerating the rate‐limiting *cis‐trans* and *trans‐cis*‐conformational changes at Xaa‐Pro peptide bonds (where Xaa is any amino acid preceding the proline residue) (Dunyak & Gestwicki, 2016; Wang & Heitman, 2005). Within the *T. gondii* genome, several *cyp* and *cyp*‐like gene sequences exist, but the roles of the corresponding proteins in the parasite are largely unknown (Krücken et al., 2009). Recently, we characterized TgCyp23 among the various Cyps of the parasite, demonstrating its high PPIase activity and its ability to bind cyclosporin A (CsA), a well‐known inhibitor of Cyps (Favretto et al., 2023). Notably, CsA exhibits antimicrobial activity against various protozoan pathogens, including *T. gondii* and *T. cruzi*, suggesting the significance of toxoplasma Cyps in a potential antitoxoplasma mechanism of CsA and their candidacy as drug targets (Chappell & Wastling, 1992; Page et al., 1995; Perrone et al., 2018). However, the immunosuppressive nature of CsA, attributed to the formation of a ternary complex between CsA, the human cyclophilin A (CypA), and the Ca^2+^‐dependent phosphatase calcineurin (Cn) (Huai et al., 2002; Liu et al., 1991; Niemoeller et al., 2006), presents substantial therapeutic challenges for its use as an antitoxoplasma drug as it would lock down the immune system.

**FIGURE 1:**
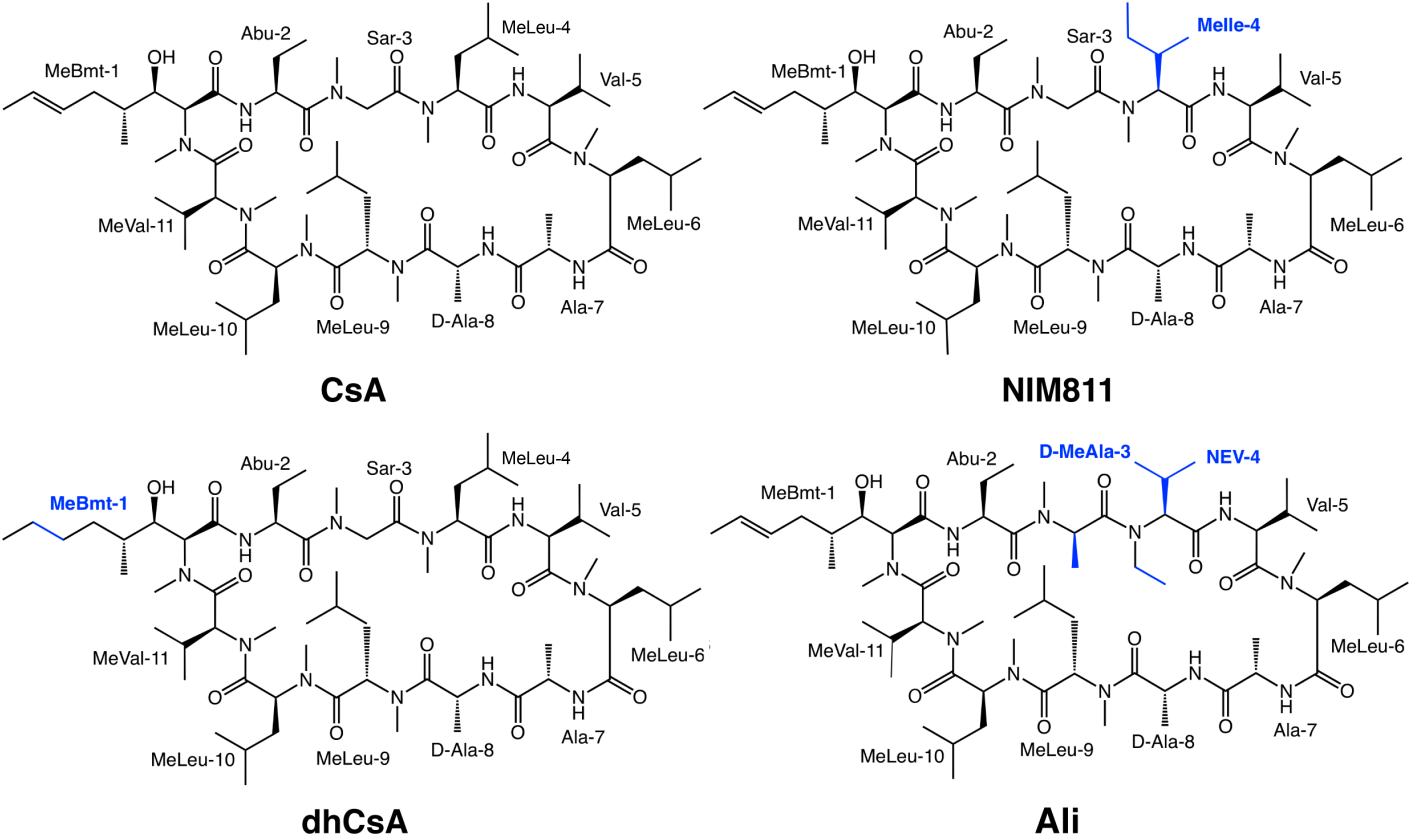
Chemical structure of Cyclosporin A (CsA), NIM811, dihydrocyclosporin A (dhCsA), and Alisporivir (Ali). Modifications are highlighted in blue.

In this study, we assess the potential of three non‐immunosuppressive CsA derivatives to target TgCyp23. The selected drugs were NIM811, Alisporivir, and dihydrocyclosporin A (Schiene‐Fischer et al., 2022). NIM811, a CsA derivative where N‐methylleucine (MeLeu‐4) is replaced by N‐methylisoleucine (MeIle‐4) at position 4 (Figure 1), retains full capacity to inhibit CypA PPIase activity with a K_i_ value of 2.1 nM but possesses significantly reduced immunosuppressive activity (Schiene‐Fischer et al., 2022; Ptak et al., 2008). The compound has shown potent antiviral activity against human immunodeficiency virus (HIV‐1) and is in phase 2 of clinical trials against hepatitis C virus (HCV) infections. Importantly, it displays strong parasiticidal effects against *T. cruzi* and *P. vivax* pathogens (Búa et al., 2008; Goto et al., 2006; Kocken et al., 1996; Ma et al., 2006).

Alisporivir (Debio 025) differs from CsA in the residues at positions 3 and 4. It replaces the sarcosine (Sar‐3) moiety at position 3 and MeLeu‐4 residue at position 4 with N‐methyl‐D‐Alanine (MeAla‐3) and N‐ethylvaline (NEV‐4), respectively (Figure 1). These positions in the ligand have been associated with its conformational flexibility, its affinity for Cyps, and its non‐immunosuppressive properties (Dujardin et al., 2018; Fu et al., 2014; Kallen et al., 1998; Zhang et al., 2004). Alisporivir is a potent inhibitor of CypA PPIase activity (K_i_ value of 0.34 nM), which has demonstrated efficacy in clinical trials for the treatment of HCV infections, HIV, and equine arteritis virus infections (Coelmont et al., 2010; de Wilde et al., 2013; Gallay & Lin, 2013; Ptak et al., 2008). Moreover, it has also proved to be effective in an animal model of Duchenne muscular dystrophy (Reutenauer et al., 2008; Schiavone et al., 2017; Tiepolo et al., 2009) and is currently undergoing clinical trials for the treatment of hospitalized patients with infections caused by SARS‐CoV‐2 (COVID‐19). Importantly, it has shown utility in targeting artemisinin‐resistant *P. falciparum* malarial parasites (Chaurasiya et al., 2022; Dujardin et al., 2018; Softic et al., 2020).

Dihydrocyclosporin A (dhCsA), a closely related co‐metabolite of CsA, presents a saturation of the double bond of the unusual amino acid (4R)‐4‐([E]‐2‐butenyl)‐4‐methyl‐N‐methyl‐(L)‐threonine (MeBmt‐1) in position 1, has minimal reported immunosuppressive activity, and serves as a control in determining the role of immunosuppression in CsA‐based treatment of parasitic infections (Figure 1) (Chappell et al., 1987; Meier et al., 1990).

In this work, the structure of the TgCyp23–ligand complexes for the three CsA non‐immunosuppressive analogs was solved by X‐ray crystallography and compared with the structure of the TgCyp23–CsA complex.

The high‐resolution X‐ray data allowed for the precise positioning of the ligands within the TgCyp23 binding pocket and provided detailed insights into the interactions between the protein and the ligands. Additionally, nuclear magnetic resonance (NMR) spectroscopy was employed to observe the interfaces of protein–ligand interactions. The NMR experiments complemented the structural data acquired from X‐ray crystallography by providing dynamic, and residue‐level insights into molecular interactions, thereby offering a more detailed and comprehensive understanding of TgCyp23–ligand complexes. The binding affinities and the inhibitory activities of the three CsA derivatives against TgCyp23 were determined and rationalized by the observed structural data. Furthermore, the impact of the drugs on the binding interaction with Cn and the resulting immunosuppressive activity were also discussed.

Overall, our findings demonstrated the tight interaction of the CsA analogs with TgCyp23 and elucidated the specific contribution of CsA chemical modifications to TgCyp23 binding, underscoring their potential therapeutic application as effective antitoxoplasma drugs.

## 2 RESULTS

### 2.1 *In silico* molecular docking

After identifying the conserved amino acid residues that form the catalytically active site of the PPIase domain of CypA (Davis et al., 2010; Mikol et al., 1993; Yang et al., 2015; Zydowsky et al., 1992), we conducted a search for structural analogs of CsA or CsA‐based inhibitors that target this region to achieve high specificity. These analogs/inhibitors were previously identified in co‐crystal structures with CypA (Dujardin et al., 2018; Fu et al., 2014; Kallen et al., 1998; Kuglstatter et al., 2011; Martin et al., 2018; Mikol et al., 1993; Mikol et al., 1994a; Mikol et al., 1994b; Papageorgiou et al., 1994; Sedrani et al., 2003; Steadman et al., 2017; Warne et al., 2016). The purpose of this workflow was to select the most promising candidates with potential inhibitory activity for TgCyp23. Therefore, the predicted binding energies and affinities from the screening procedure were intended to provide a ranking among putative inhibitors.

*In silico* analysis was performed by docking each molecule onto the crystal structure of TgCyp23 (see Experimental Section). The binding energy (kcal/mol) and predicted binding affinity (nM) of the top 24 ligands are reported in Table S1. Among the high‐scoring compounds, we selected for further experimental characterization, those meeting the following criteria: (i) possessing a structure similar to CsA, (ii) demonstrating antiparasitic activity, (iii) showing a predicted high affinity comparable to CsA (Table S1), and (iv) having established non‐immunosuppressive properties. Accordingly, the selected drugs were NIM811 (MeIle‐4‐CsA), Alisporivir (D‐MeAla‐3‐EtVal‐4‐CsA), and dhCsA (Búa et al., 2008; Chaurasiya et al., 2022; Kocken et al., 1996; Zheng et al., 2020). Notably, NIM811 and Alisporivir are advanced host‐targeting antiviral agents in clinical development.

### 2.2 Structure of the TgCyp23–analogs complexes

**FIGURE 2:**
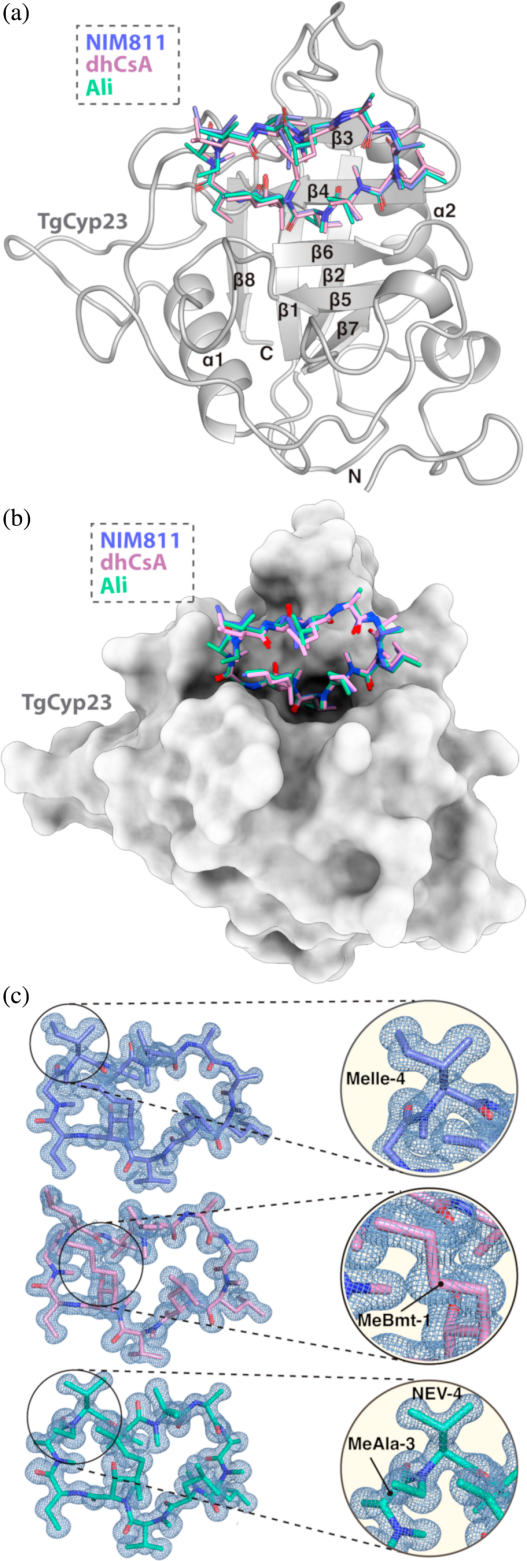
Crystallographic structures of TgCyp23‐ligand complexes. (a) Overall structure of TgCyp23 complexes. The TgCyp23 protein (as observed in chain B of the TgCyp23:dhCsA complex) is shown in gray cartoon while NIM811, dhCsA, and Alisporivir are represented in sticks (purple, pink, and cyan, respectively). N indicates the N‐terminus region and C the C‐terminus. Secondary structural elements are labeled. (b) Structural superimposition of the three non‐immunosuppressive compounds bound to TgCyp23. The TgCyp23 protein (as observed in chain A of the TgCyp23:NIM811 complex) is shown as a gray surface, NIM811, dhCsA, and Alisporivir are represented in sticks (purple, pink, and cyan, respectively). (c) 2Fo‐Fc electron density maps observed for the three ligands. Maps are contoured at 1σ. A detailed view of each ligand's modification compared to CsA is shown on the right side following the same color's legend.

The crystal structure of TgCyp23:CsA (PDB 8B58) was previously reported and revealed conservation in the folding pattern with other Cyps (Favretto et al., 2023). TgCyp23 exhibits the typical eight‐stranded antiparallel β‐barrel structure, with two α‐helices flanking the barrel on either side, all of them connected by loops (Figure 2a). The main difference in TgCyp23 is related with the 40 extra residues in N‐terminal region compared to human CypA. Here, three new structures of the protein in complex with the three non‐immunosuppressive derivatives NIM811, dhCsA, and Alisporivir have been solved at 1.17, 1.20, and 1.20 Å resolution, respectively. All these structures were solved by the molecular replacement method, using one TgCyp23 monomer as the searching model (see Experimental Section and Table S2). In all cases, the asymmetric unit is composed of two monomers with almost identical structure (RMSD values between 0.112 and 0.141 Å for the Cα superpositions). NIM811, dhCsA, and Alisporivir are attached to the CsA binding pocket (Figures 2a,b and S1). The high quality of the electron density maps allowed perfect placement of the ligands into the TgCyp23 binding site as well as precise identification of the subtle modifications defining each of the three non‐immunosuppressive CsA derivatives (Figure 2c). The backbone for these three derivatives is nearly identical to that of CsA. Remarkably, all the modifications in the non‐immunosuppressive derivatives are exposed, protrude from the binding site, and are not involved in the crystal packing.

### 2.3 Interaction patterns of CsA analogs with TgCyp23

The TgCyp23:CsA interaction has been previously described (Favretto et al., 2023). CsA is located in a pocket built by β3, β4, and the loops Lβ5β6 (connecting β5➔ β6) and Lβ6β7 (connecting β6➔ β7) as shown in Figure 2a. The binding pocket is surrounded by the residues Arg99, Phe104, Met105, Ile106, Gln107, Gly116, Ala145, Asn146, Ser147, Gln155, Phe157, Trp165, Leu166, and His170. Importantly, six out of the eleven CsA residues present interactions with the protein (Figure S1a). The stabilization of CsA is primarily performed through polar interactions and Van der Waals forces. Specifically, a total of eight hydrogen bonds are established between CsA and TgCyp23 (Table S3, Figure S1a).

**FIGURE 3:**
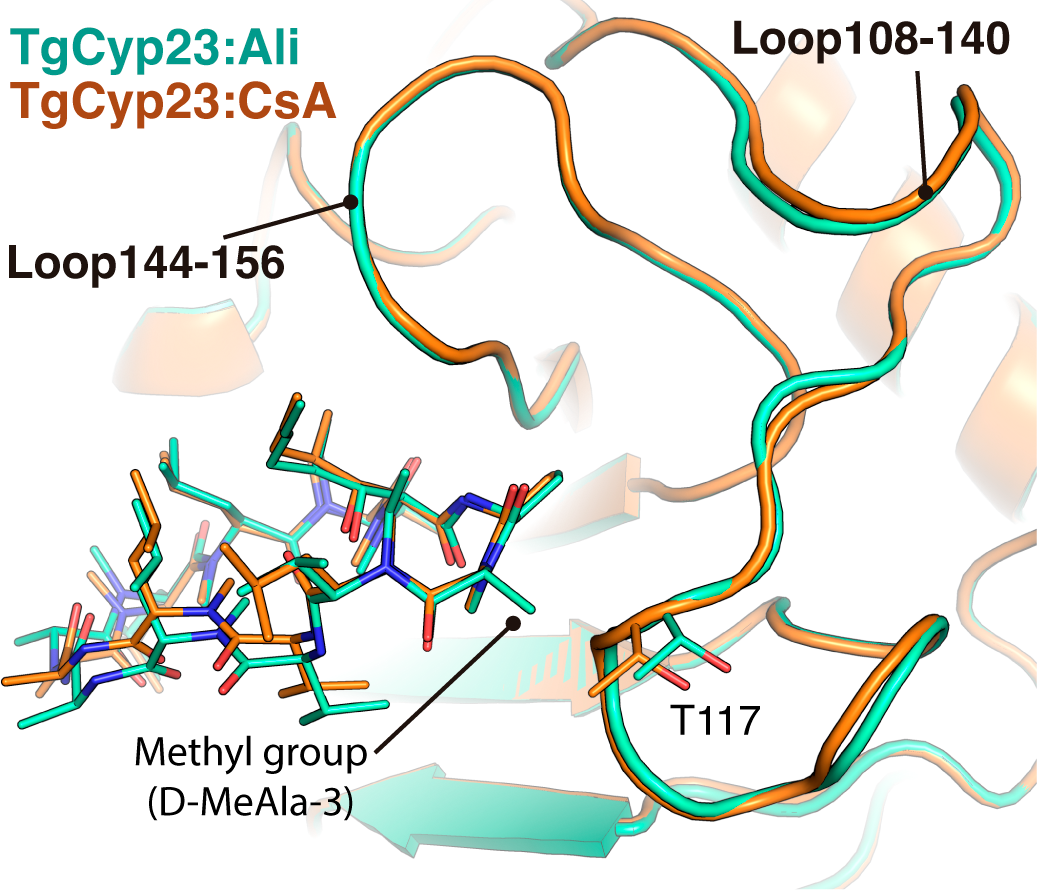
Comparison of loops L108–140 and L144–156 between TgCyp23:CsA and TgCyp23:Alisporivir complexes. Both structures are aligned and displayed in cartoon, TgCyp23:CsA in orange and TgCyp23:Ali in cyan. CsA and Alisporivir are also displayed in orange and cyan sticks, respectively. Thr117 is labeled with one‐letter code.

The detailed analysis of the three derivative complexes, TgCyp23:dhCsA, TgCyp23:NIM811, and TgCyp23:Alisporivir, compared to TgCyp23:CsA (PDB: 8B58) reveals nearly the same interaction pattern (Figure S1b–d), with only very small differences in hydrogen bond distances (Table S3). Also, small differences are observed in the loops L108–140 and L144–156 (Figure 3), in which we found some residues with double side‐chain conformations. It is worth mentioning that these two loops present the largest chemical shift perturbation difference between the TgCyp23:CsA and TgCyp23 in complex with CsA analogs (see below). Remarkably, in the TgCyp23:Alisporivir complex, a new Van der Waals interaction between the two methyl groups from D‐MeAla‐3 (Alisporivir) and Thr117 (TgCyp23) is observed (red dash line in Figure S1d). Thr117 exhibits a displacement of 1 Å in the carbon backbone, a phenomenon anticipated due to Alisporivir's additional methyl group at position 3. This alteration induces a slight shift in the L108–140 loop, with the most notable effect observed in the residue Thr117, the nearest amino acid to the methyl group (Figure 3).

### 2.4 NMR analysis of binding interfaces

The analysis of protein–ligand interactions using NMR spectroscopy provided further insights into the amino acids forming the binding interface of TgCyp23 with the three analogs in solution.

Standard three‐dimensional experiments were conducted on the double‐labeled ^15^N‐^13^C‐TgCyp23 to assign the backbone of TgCyp23 in both its unbound and drug‐bound forms, thereby refining the definition of the binding interface. All assignments covered over 90% of the backbone resonances.

**FIGURE 4:**
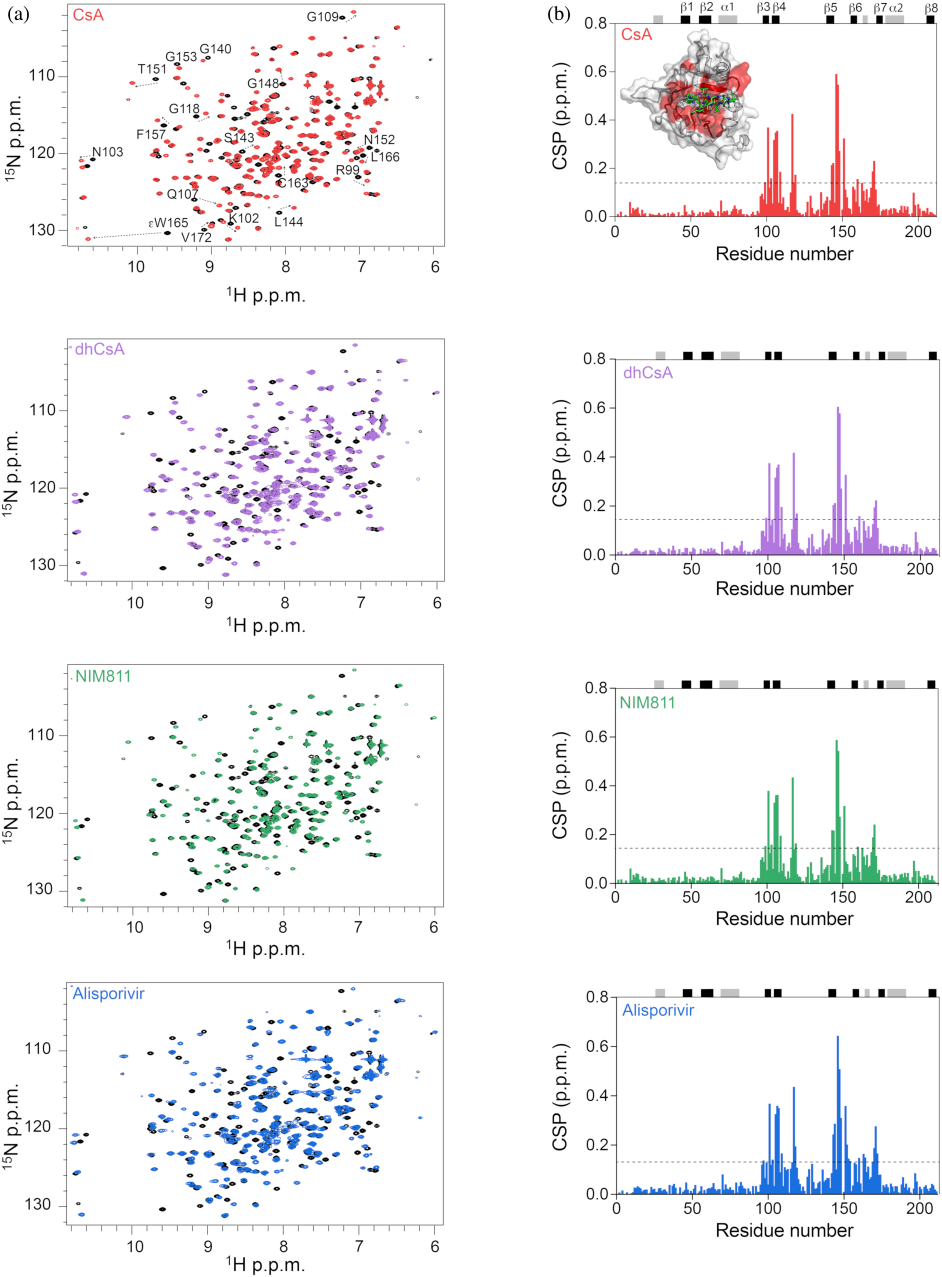
NMR analysis of the interaction between TgCyp23 and CsA analogs. (a) ^1^H‐^15^N‐HSQC spectra of TgCyp23 in the absence (black) and in the presence of a two‐fold molar excess of CsA (red), dhCsA (violet), NIM811 (green), and Alisporivir (blue). Selected peaks are indicated. (b) Residue specific chemical shift perturbation (CSP) analysis of the interaction observed upon binding of the three compounds to ^15^N‐TgCyp23. Secondary structure elements are shown on the top of the panels (α‐helix in gray, β‐sheets in black). The color code is as in panel (a).

Next, ^1^H‐^15^N‐labeled TgCyp23 samples were mixed with increasing amounts of each unlabeled drug compounds, and the resulting ^1^H‐^15^N‐HSQC spectra were recorded and used to map those residues that are in direct contact with the ligands (Figure 4a). Upon ligand binding, several cross peaks in the ^1^H‐^15^N‐HSQC spectrum of ^15^N‐TgCyp23 exhibited significant intensity loss and notable chemical shift changes (CSP >0.15 p.p.m.) (Figures 4a and S2). Such pronounced changes in chemical shifts were already detected by NMR spectroscopy for the complexes formed not only by human CypA with CsA and Alisporivir (Favretto et al., 2020; Landrieu et al., 2010), but also for other PPIases upon titration with the immunosuppressive ligands (Medek et al., 2000). Throughout the titration process, we observed several resonances corresponding to the unbound state progressively disappearing and reappearing in another part of the spectrum (bound state), suggesting that the binding occurred slowly on an NMR time scale. This is consistent with the formation of a high‐affinity complex in the nanomolar range (see below). A quantitative analysis of the CSP was performed and plotted against the residue number (Figure 4b) with the TgCyp23:CsA complex serving as a reference. All the compounds induced a similar CSP perturbation profile within the ^1^H‐^15^N‐HSQC of TgCyp23, with changes mainly localized in the β3 − β6 sheet region and in the loops connecting the sheets that correspond to the active site of TgCyp23. This suggests that all the ligands bind within the catalytic cavity, in agreement with the conservation of inhibitor–protein interactions observed for the three non‐immunosuppressive CsA derivatives in the crystallographic complexes.

**FIGURE 5:**
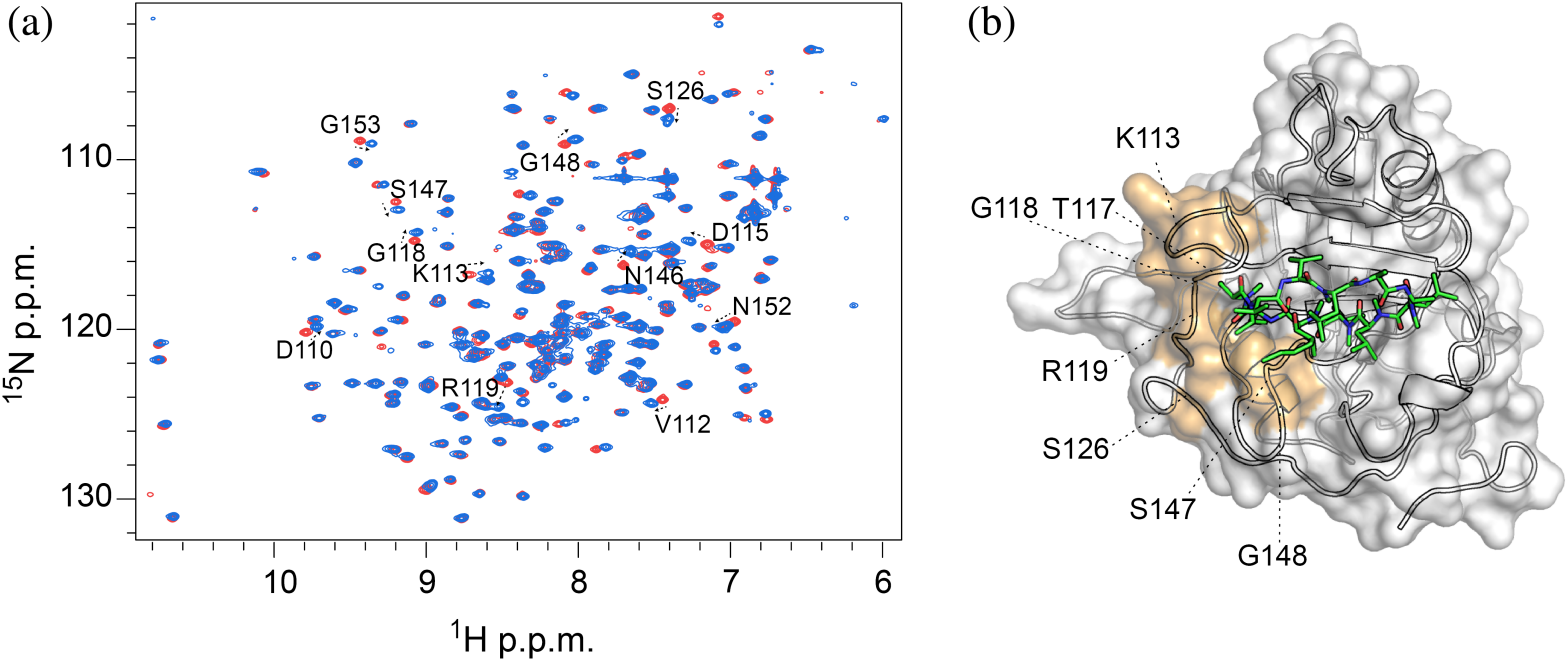
Chemical shift perturbation difference between TgCyp23 in complex with CsA or Alisporivir. (a) Superposition of ^1^H‐^15^N‐HSQC spectra of TgCyp23:CsA (red) and TgCyp23:Alisporivir (blue). (b) Mapping of the chemical shift perturbation analyzed in panel (a) on TgCyp23 structure in complex with Alisporivir. The most affected residues are reported in orange.

To explore further the impact of the chemical modifications of CsA that lead to the observed differential chemical shifts, we also calculated the CSP difference between TgCyp23 in complex with CsA and the analogs (Figures 5a and S3). The residues most significantly affected by the binding were then mapped onto the 3D structure of TgCyp23 (PDB: 8B58) (Figure 5b). Remarkably, both NIM811 and dhCsA closely resembled the TgCyp23:CsA complex, implying that chemical modifications at position 4 of CsA exert negligible influence on the binding to the protein. Conversely, in agreement with structural rearrangements shown in Figure 3, significant chemical shift perturbations (CSP >0.05 p.p.m.) were evident in the TgCyp23:Alisporivir complex, particularly in residues Gly109, Asp110, Phe111, Val112, Lys113, Asp115, Thr117, Gly118, Arg119, Ser126, and residues Leu144, Asn146, Ser147, Gly148, Asn152, Gly153, and Cys154, with the sole exception of Val172, situated on the opposing side (Figure 5, Figure S3).

### 2.5 Drug binding and inhibition activity

**FIGURE 6:**
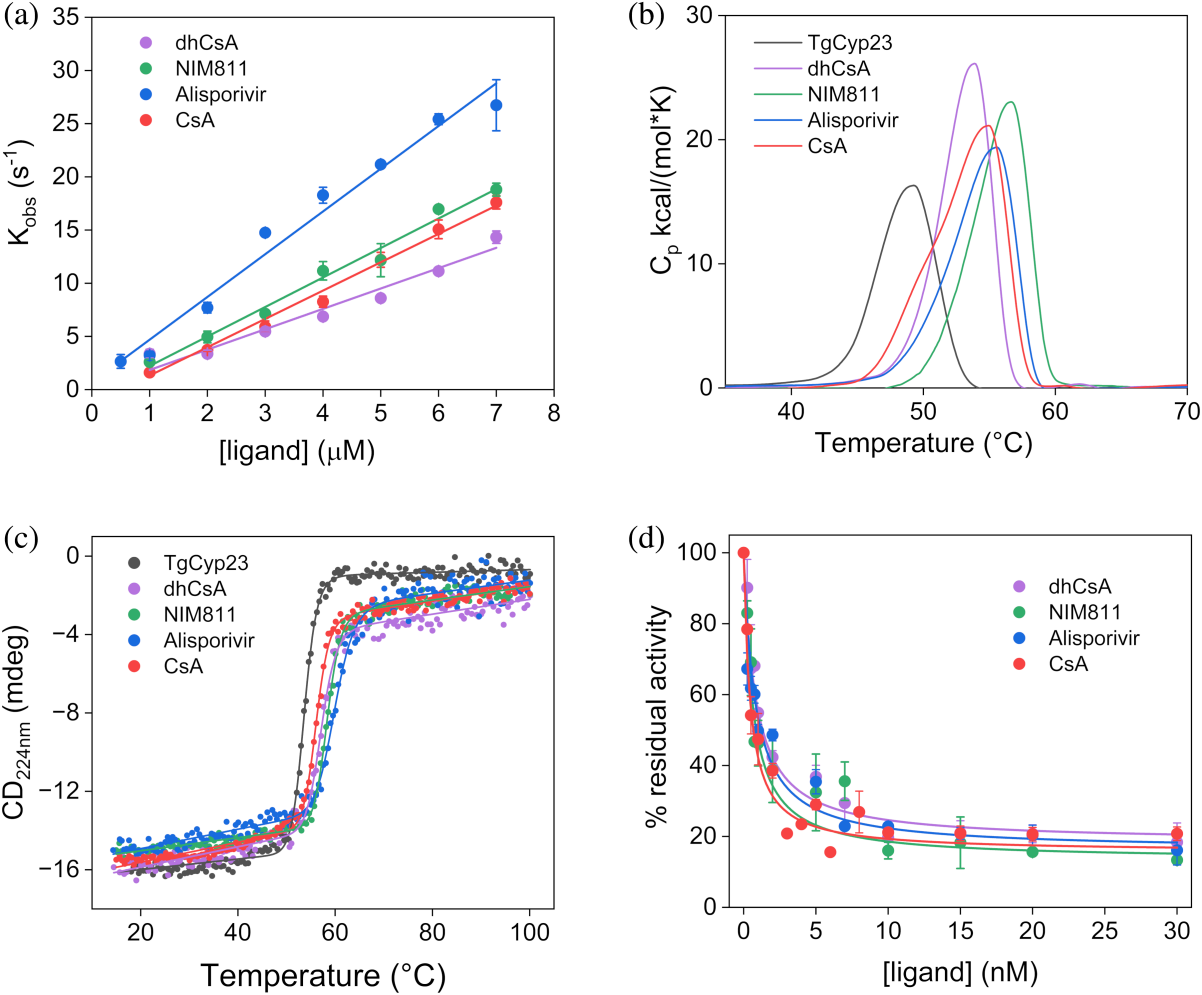
Effects of CsA analogs on TgCyp23 properties. (a) Pseudo‐first order kinetics of the binding reaction between TgCyp23 versus different concentrations of CsA and CsA analogs. (b) DSC data for denaturation of TgCyp23 in the absence and presence of CsA analogs. (c) CD thermal denaturation profiles of TgCyp23 in the absence and presence of CsA analogs. (d) PPIase activity of TgCyp23 in the presence of increasing amounts of CsA analogs. The color code in all panels is as follows: TgCyp23 alone (black), TgCyp23 in the presence of CsA (red), NIM811 (green), Alisporivir (blue), and dhCsA (violet).

The differences in TgCyp23–ligand interactions were further elucidated by assessing the binding affinities of the target protein for the three drugs using isothermal titration calorimetry (ITC) (Figure S4). Each compound exhibited an exothermic binding profile with TgCyp23 characterized by a singular event consistent with a 1:1 ligand:protein stoichiometry. The protein demonstrated nanomolar binding affinity with all the drugs (Table 1). Specifically, Alisporivir displayed the highest interaction affinity with TgCyp23 (K_d_ = 15 ± 3 nM) while dhCsA showed the lowest affinity (K_d_ = 200 ± 92 nM) (Figure S4 and Table 1). Of note, the affinity of Alisporivir surpassed that of CsA (K_d_ = 82 ± 15 nM) (Favretto et al., 2023). Accordingly, the association rate constant (k_on_) for TgCyp23‐Alisporivir is higher than that for the TgCyp23–CsA complex as determined by stopped flow rapid kinetics (Figure 6a and Table 1).

**TABLE 1:** Biochemical characteristics of TgCyp23 in the absence and presence of CsA and its derivatives.

	K_d_ (ITC) (nM)	k_on_ (μM^−1^ s^−1^)	T_m_ (CD) (°C)	T_m_ (DSC) (°C)	IC_50_ (nM)
TgCyp23			52 ± 1	49 ± 1	
+ CsA	82 ± 15	2.69 ± 0.12	55 ± 1	54 ± 1	0.6 ± 0.1
+ dhCsA	200 ± 92	1.85 ± 0.11	57 ± 1	53 ± 1	1.2 ± 0.2
+ NIM811	43 ± 10	2.77 ± 0.15	58 ± 1	56 ± 1	0.85 ± 0.03
+ Alisporivir	15 ± 3	3.97 ± 0.21	59 ± 1	55 ± 1	0.77 ± 0.01

The binding of all three compounds also had a significant impact on the thermal stability of the protein, leading to an overall increase in the melting temperature (T_m_) of TgCyp23, as observed through both differential scanning calorimetry (DSC) and circular dichroism (CD) analysis (Figure 6b,c and Table 1). This effect consistently correlated with the higher binding affinity of Alisporivir and NIM811 to TgCyp23 compared to dhCsA, as determined by ITC analysis.

The inhibitory effect of the PPIase activity of TgCyp23 of the selected compounds was assessed by the PPIase assay utilizing a previously established protocol (Favretto et al., 2023). All three compounds inhibit the catalytic activity of TgCyp23, with IC_50_ values between 0.8 and 2 nM, with Alisporivir having the highest inhibitory activity (Figure 6d, Table 1). These values closely align with those of CsA as previously determined through the chymotrypsin‐coupled peptidyl‐prolyl isomerization assay (Favretto et al., 2023).

### 2.6 Structural impact of CsA analogs in the formation of a ternary complex with the human CypA, and the human calcineurin

**FIGURE 7:**
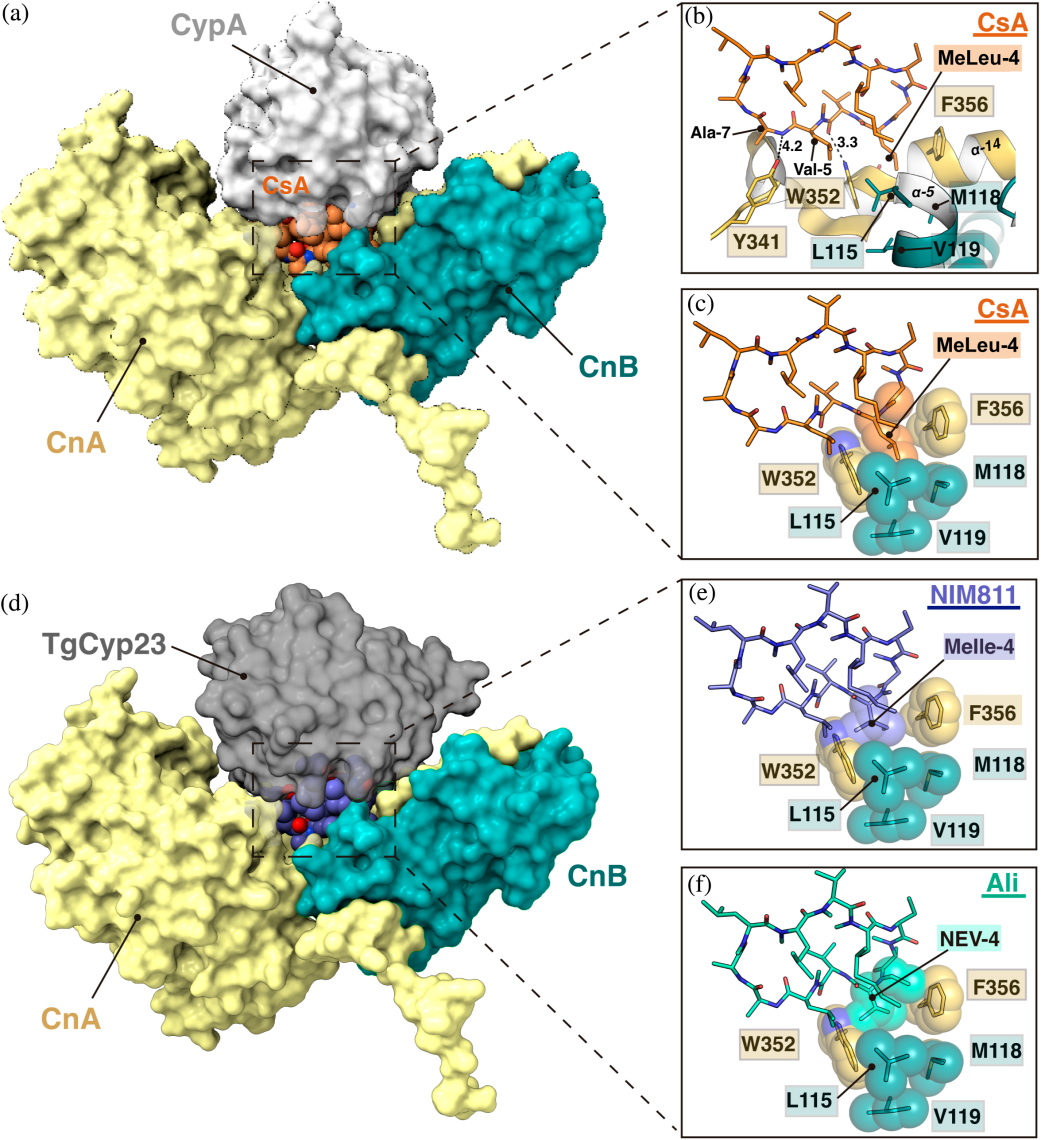
Interaction basis of CypA‐CsA‐Calcineurin human complex (PDB code 1M63) and potential impact of CsA analogs. (a) Assembly of the human ternary complex, proteins CypA, CnA, and CnB are displayed as a surface (gray, yellow, and blue respectively). CsA is presented in orange spheres. (b, c) Hydrophobic pocket of Cn (residues from CnA and CnB in yellow and blue sticks/spheres, respectively). MeLeu‐4 (CsA), in orange sticks/spheres, is tightly fitted into the cavity. (d) TgCyp23:NIM811 complex superposition onto the ternary Cn–CypA–CsA human complex (CsA and CypA have been omitted for clarity reasons). (e) Predicted clashes between MeIle‐4 (NIM811), and Trp352 (CnA). NIM811 is depicted in purple sticks. (f) Predicted clashes between NEV‐4 (Alisporivir), Trp352 (CnA), and Phe356 (CnA). Alisporivir is painted in cyan sticks.

CsA binds to human CypA, forming a heterodimeric complex that inhibits Cn, a serine/threonine phosphatase involved in the immune response pathway, leading to immunosuppression. The crystal structure of the ternary Cn–CypA–CsA human complex (PDB code 1M63) has been previously described (Huai et al., 2002), and it is displayed in Figure 7a. CsA (orange spheres) is positioned in the middle of CypA (in gray surface) and Cn, comprising two protein chains, CnA and CnB (in yellow and blue surfaces, respectively). Van der Waals forces primarily stabilize the CsA‐Cn binding, with few hydrogen bond interactions observed between Ala‐7 and Val‐5 (from CsA) and Tyr341 and Trp352 (from CnA) (Figure 7b). Thus, Cn presents a hydrophobic cavity into which the residue MeLeu‐4 of CsA is inserted (Figure 7c). This cavity is delimited by the residues Trp352, Phe356 (from α14 of CnA), and Leu115, Met118, and Val119 (from α5 of CnB) (Figure 7c).

TgCyp23 shows remarkable sequence identity with human CypA (~52% of identity) but has a 40‐aminoacid N‐terminal extension. The superposition between them presents an RMSD of 0.494 Å (Figure S5a). Besides, CsA is placed exactly in the same cavity in both Cyps (Figure S5b,c). We reasoned that, as the interactions between CsA analogs and TgCyp23 are nearly identical to that of CsA (Figure S1), the lack of immunosuppressing activity of CsA analogs could come from differences in the interaction with human Cn. With this purpose, we superimposed our TgCyp23:NIM811and TgCyp23:Ali complexes onto the Cn–CypA–CsA complex (Figure 7d). This comparison provides insights into the structural details that could be responsible for the lack of immunosuppressive activity of CsA analogs. The MeLeu‐4 residue of CsA is replaced by MeIle‐4 in NIM811 (Figure 1). This amino acid has a bulkier side chain that could provoke clashes with the Trp352 residue of the cavity (Figure 7e). In Alisporivir, Sar‐3 and MeLeu‐4 of CsA are replaced by MeAla‐3 and NEV‐4, respectively (Figure 1). Similarly to NIM811, in Alisporivir, the side chain of NEV‐4 creates steric hindrance, preventing it from occupying the hydrophobic Cn cavity and proper interactions. Moreover, the N‐ethyl group would also provoke clashes with Phe356 residue (Figure 7f). Thus, our model provides a plausible explanation to understand why CsA analogs could act as antimicrobials against Toxoplasma, but without blocking the immune response in humans.

## 3 DISCUSSION

Toxoplasmosis is a globally widespread disease, yet existing chemotherapy options exhibit limited efficacy and safety, often associated with parasite resistance and significant adverse effects that hinder patient compliance. Therefore, there is an urgent demand for the discovery of novel, effective drug candidates and therapeutic targets in combating this disease. Repurposing existing drugs as novel antitoxoplasmosis agents provides a promising alternative in drug discovery. In this study, we demonstrate the potential of the non‐immunosuppressive CsA analogs NIM811, Alisporivir, and dhCsA in targeting the toxoplasma TgCyp23 protein *in vitro*.

CsA is a widely recognized cyclic peptide employed as an immunosuppressant to prevent organ rejection in transplantation. However, it also exhibits antiparasitic activity, even if its mechanism of action in parasites remains poorly understood at the molecular level. CsA targets Cyps and inhibits their PPIase activity with a dissociation constant in the lower nanomolar range (Walsh et al., 1992). However, immunosuppression is not directly linked to this inhibitory mechanism. Instead, the molecular interface resulting from the CypA‐CsA binding enables the complex to inhibit the phosphatase Cn, crucial for T‐cell activation. The structure elucidation of the CypA‐CsA‐Cn ternary complex (Huai et al., 2002) revealed the specific interactions of CsA with CypA and Cn.

CsA derivatives that avoid immunosuppression maintain or enhance binding to CypA while reducing affinity toward Cn. Effective structural modifications for mitigating immunosuppression involve substituting amino acids at positions 3 and 4 of CsA, as demonstrated by the antiviral agents NIM811 and Alisporivir (Figure 1) (Landrieu et al., 2010; Rosenwirth et al., 1994). CsA derivatives with modifications at residue 1 (such as dhCsA, which is a co‐metabolite of CsA used as a control to determine the role of CsA in pharmacology, Figure 1) have also shown decreased immunosuppressive activity without compromising Cyp affinity, even though the MeBmt‐1 side chain is positioned in the part of CsA involved in the binding of Cyp (Aebi et al., 1990; Mikol et al., 1993; Rich et al., 1989).

The high‐resolution X‐ray crystal structures of TgCyp23 in complex with the three CsA derivatives, NIM811, Alisporivir, and dhCsA, reported here demonstrate that the ligands are well‐suited to enable strong interactions with the catalytic site of TgCyp23, closely resembling the interactions between CsA and TgCyp23 (Favretto et al., 2023), with all the modifications exposed and protruding from the binding site. The observed conservation of NMR chemical shift between the CsA derivatives in complex with TgCyp23 further supports the conservation of the global conformation and binding mode for the analogs. This corroborates the functional observations that all three compounds behave biochemically like CsA, effectively binding to TgCyp23 with nanomolar affinity, stabilizing the enzyme, and inhibiting its PPIase activity with low nanomolar IC_50_ values.

Remarkably, Alisporivir emerged as the CsA derivative with the highest binding affinity (K_d_ = 15 nM) and high inhibitory potency (IC_50_ = 0.7 nM) against TgCyp23, consistent with previous reports for human CypA in its complex with CsA or Alisporivir (Launay et al., 2013). Comparison of the CsA and Alisporivir complexes structures allowed us to rationalize the higher affinity of Alisporivir, revealing subtle effects due to the introduction of N‐methyl group at position 3. This modification induces the formation of superior Van der Waals contacts between the unique side chain of the ligand and TgCyp23 (T117 residue) and causes protein structural rearrangements, specifically within loops L108–140 and L144–156. The TgCyp23–Alisporivir interface defined on the basis of the obtained high‐resolution structure is consistent with the NMR chemical shift perturbations observed for ^15^N‐labeled TgCyp23 upon binding of Alisporivir. Indeed, the direct interaction of some residues within the L108–140 loop of TgCyp23 with the methyl group on residue 3 of Alisporivir explains the significant differential chemical shifts observed in the NMR spectra. For example, both Thr117 and Arg119 exhibit significant CSP of 0.07 and 0.2 ppm, respectively. These residues are in tight contact with the methyl group of Alisporivir in position 3 and directly interact with it (Thr117) or experience significant rearrangement of their side chains (Arg119) due to the formation of hydrogen bonds distinct from those in the TgCyp23:CsA complex. Further chemical shift perturbations in the L144–156 loop may be attributed to conformational rearrangements and/or changes in dynamics induced by the introduction of the methyl group at position 3 of CsA. Our findings closely align with the CSP profile previously reported between the CypA:CsA and CypA:Alisporivir complexes (Dujardin et al., 2018; Landrieu et al., 2010), which reveal significant differences within the L63–76 loop of CypA (corresponding to the region Gln107‐Leu120 in TgCyp23) and the L101–112 loop (corresponding to Ala145‐Phe156 portion in TgCyp23).

Position 4 in CsA derivatives plays a pivotal role in their immunosuppressive properties (Fu et al., 2014; Huai et al., 2002; Kallen et al., 1998). Specifically, Cn tightly binds with the side chain at position 4, wherein the MeLeu side chain of CsA engages in tight aromatic interactions with the Trp352 and Phe356 residues of the catalytic subunit of Cn (Huai et al., 2002). It has been demonstrated that the presence of a Cβ‐branched residue at position 4 is sufficient to disrupt the interaction with Cn, consequently reducing the immunosuppressive activity. Notably, NIM811 and Alisporivir, featuring MeIle and NEV residue at position 4, respectively, lack immunosuppressive effects (Coelmont et al., 2010; Ma et al., 2006; Paeshuyse et al., 2006). Superimposition of the TgCyp23:Alisporivir and TgCyp23:NIM811 complexes onto the structure of the CypA–CsA–CN ternary complex (Huai et al., 2002) clearly reveals that the side chain of MeIle‐4 in NIM811 and NEV‐4 in Alisporivir cannot enter the Cn hydrophobic cavity to establish a proper interaction. We observed a steric clash with the Trp352 aromatic ring that constitutes one wall of the Cn pocket. Moreover, the N‐ethyl group of Val‐4 points toward the same Cn cavity, causing clashes with Phe356 residue. The MeIle‐4 side chain of NIM811 and NEV‐4 side chain of Alisporivir prevent the TgCyp23–drug complex from interacting with Cn. Hence, this likely offers a plausible explanation for why CsA analogs can function as antimicrobials against Toxoplasma without inhibiting the human immune response.

In conclusion, the discovery of the potent inhibitory effects of the non‐immunosuppressive antiviral agents NIM811 and Alisporivir on a Toxoplasma Cyp suggests their potential future use, either alone or in combination with existing antitoxoplasmosis agents. This positions them as promising and potent candidate drugs against Toxoplasma parasites in the context of drug repurposing. However, since the Toxoplasma genome contains multiple *cyp* genes, it is conceivable that these drugs bind to other Cyps, which also contribute to the PPIase activity. To further substantiate our findings, future comprehensive investigations into the prospective antitoxoplasmosis properties of these drugs should be conducted using TgCyp23 mutated and/or disrupted parasites.

## 4 EXPERIMENTAL SECTION

### 4.1 Materials

All chemicals were purchased from Sigma‐Aldrich unless otherwise noted. CsA, Alisporivir, and NIM811 were purchased from MedChemExpressEurope, while dhCsA was obtained from Hölzel Diagnostika Handels GmbH. All compounds were >95% pure by HPLC or LCMS.

### 4.2 Protein production

TgCyp23 was expressed and purified as described in (Favretto et al., 2023). Labeled TgCyp23 for nuclear magnetic resonance (NMR) experiments was prepared in M9 minimal medium supplemented with^15^NH_4_Cl (1 g/L), or ^15^NH_4_Cl (1 g/L) and [^13^C] glucose (4 g/L).

### 4.3 *In silico* analysis

Several docking suites are available for protein–ligand docking. YASARA (Krieger & Vriend, 2014) is a robust and versatile software suitable for molecular modeling, dynamics, and docking. Autodock VINA (Trott & Olson, 2010) is one of the most reliable and widely used algorithms by researchers globally. YASARA‐embedded docking approaches, Autodock (Morris et al., 1998) and Autodock VINA, are gold standard algorithms for predicting protein–ligand complexes. We selected VINA for our screening because it handles large ligands more efficiently and has faster computing times compared to Autodock. Additionally, before docking all the compounds to TgCyp23, we validated the software by disassembling the crystal structure of the CypA–CsA complex and performing docking on apo‐CypA. The structural alignment showed an RMSD of only 0.8 Å between the experimental and predicted pose for CsA, confirming the algorithm's accuracy in predicting protein–peptide complexes.

Molecular docking was performed using YASARA (Krieger & Vriend, 2014) Structure, version 23.9.9, operating under Windows 11 Pro an HP OMEN 880‐102 nL workstation (Intel Core i7‐8700K, 2.8 GHz CPU, and 1 GB memory). The crystallographic structure of CypA and CsA complex (PDB code 1CWA (Mikol et al., 1993)) was retrieved from the Protein Data Bank (RCSB). The TgCyp23 structure was structurally aligned to the CsA–CypA complex to build a simulation cell extended 10 Å on each side around the structure of CsA, replicating the same binding site of CypA on TgCyp23. The structures of CypA and CsA were then removed from the scene and the file saved as “receptor.” The 3D structure of structural analogs (Dujardin et al., 2018; Fu et al., 2014; Kallen et al., 1998; Kuglstatter et al., 2011; Martin et al., 2018; Mikol et al., 1993; Mikol et al., 1994a; Mikol et al., 1994b; Papageorgiou et al., 1994; Sedrani et al., 2003; Steadman et al., 2017; Warne et al., 2016) of CsA was downloaded from the RCSB databank as co‐crystal structures complexed with Cyp. Each complex was deprived of the protein molecules and saved as a single .pdb file to be used for *in silico* analysis. If the ligands were not available in RCSB, the.sdf files were downloaded from Pubchem (Kim et al., 2022), energy minimized with YASARA and saved as .pdb files. Docking of each ligand to the “receptor” file was carried out using the YASARA‐embedded Autodock VINA algorithm (Trott & Olson, 2010) by using the “dock_run.mcr” macro exploiting 250 runs. The YASARA macro performs structure preparation of the ligand, docking to the receptor, and results sorting by binding energy/predicted binding affinity. In YASARA, results are ranked by binding energy, where more positive energies indicate stronger binding and negative energies equate to no binding (Gao et al., 2018).

### 4.4 Crystallization of the protein–ligand complexes

For crystallization, the enzymes were buffer exchanged into 50 mM HEPES, 150 mM NaCl, 0.1 mM DTT pH 8.0. TgCyp23 was concentrated to 26 mg/mL using a centricon‐10 device. Protein was incubated with NIM811, Alisporivir, and dhCsA in powder in a molar ratio 1:6, 1:5, and 1:8 respectively, and stirred during 3 days at 4°C. The solution was then filtered through a 0.25‐μm filter to remove the excess solid. The crystallization was carried out using sitting drop vapor‐diffusion technique. Crystals grew up at 18°C after 3 days in a precipitant solution that contains 0.1 M citric acid pH 3.5 and 25% polyethylene glycol 3350.

Crystal size was further optimized by exploring the molar ratio between the protein solution (26 mg/mL) and the precipitant solution. Best crystals of NIM811, Alisporivir, and dhCsA complexes were obtained using a volume ratio of 1, 2, and 3 μL of protein against 1 μL of precipitant, respectively. Finally, crystals were dipped into a cryoprotectant buffer composed of the reservoir buffer supplemented with 25% glycerol and then flash‐frozen in liquid nitrogen.

### 4.5 Data collection and structural determination of the protein–ligand complexes

Diffraction datasets were collected in the XALOC beamline at the ALBA synchrotron (Barcelona, Spain) using a Pilatus 6 M detector and a fixed wavelength of 0.97926 Å. Collected images were processed using XDS (Kabsch, 2010) and scaled using AIMLESS from the CCP4 package (Winn et al., 2011). Crystals diffracted from 1.17 to 1.20 Å resolution and belonged to the monoclinic P 2_1_ space group. There are two monomers in the asymmetric unit with a solvent percentage between 41.4 and 43.5%. The structural determination of complexes was performed by molecular replacement method with Phaser MR of CCP4, using the known structure of TgCyp23 (PDB: 8B58). Then the corresponding ligands were manually placed using Coot (Emsley et al., 2010), and all the structures were refined with REFMAC5 (Murshudov et al., 2011) and PHENIX (Afonine et al., 2012) programs. The refinement step was also performed by calculating TLS groups and individual anisotropic B‐values. All statistics for data processing and refinement are shown in the supplemental material in Table S2.

### 4.6 Nuclear magnetic resonance experiments

NMR spectroscopy‐based studies were acquired at 298 K at a proton Larmor frequency of 600 MHz on a Bruker AVANCE NEO spectrometer, equipped triple resonance TCI cryogenic probe (Bruker). All the experiments were performed at 298 K and processed using Topspin v 3.6.2 (Bruker) and NMRpipe v.9.9 and analyzed with ccpnmr Analysis 2.5.2.

Protein backbone resonance assignment of double labeled ^15^N‐^13^C‐TgCyp23 at a concentration of 1 mM in absence or presence of the three ligands was achieved by acquiring standard 3D experiments for sequential backbone assignment (HNCA, HNCACB, HNCO, HNCACO). 3D spectra were acquired with a complex data points matrix 2048 (H) × 100 (N) × 100 (C), using the 20% of randomly spread points (20% NUS), a recycle delay of 1.2 s, and 8–16 transients. The experiments were processed using the Sparse multidimensional iterative lineshape‐enhanced (SMILE) reconstruction algorithm integrated in NMRpipe. The assignment was confirmed acquiring a 3D ^15^N‐edited NOESY‐HSQC (mixing time: 120 ms).

Protein binding to CsA and its analogs (i.e., dhCsA, NIM811, and Alisporivir) was monitored by NMR spectroscopy acquiring 2D ^1^H‐^15^N‐HSQC spectra on the uniformly labeled ^15^N‐TgCyp23 at increasing concentrations of the compound. The compounds were dissolved in a buffer containing 100% ethanol and ^15^N‐TgCyp23 was titrated with the following protein: ligand molar ratios: 1:0.2, 1:0.5, 1:1, 1:1.5, 1:2. During the titration, the final concentration of ethanol was kept ≤2.5%. ^1^H‐^15^N‐HSQC spectra were acquired after each titration step with 16 transients and 2048 (H) × 256 (N) complex points, with a recycle delay of 1.2 s.

The chemical shift perturbation of protein amide resonances between the unbound and bound states of the protein was calculated from the following equation:

$$∆\delta_{\mathrm{HN}}=\sqrt{\frac{{\left(∆\delta_{H}\right)}^{2}+{\left(\frac{∆\delta_{N}}{5}\right)}^{2}}{2}}$$

where Δδ_H_ and Δδ_N_ are the observed chemical shift changes of the amidic nuclei ^1^H and ^15^N upon ligand binding.

### 4.7 Isothermal titration calorimetry

Isothermal titration calorimetry (ITC) experiments were performed on a PEAQ‐ITC instrument from MicroCal (Northampton, MA) as previously described (Bombardi et al., 2022; Conter et al., 2021; Favretto et al., 2023). Stock solutions of dhCsA, NIM811, and Alisporivir were prepared in 100% EtOH and successively diluted in a buffer of 50 mM HEPES, pH 8.0, 100 mM NaCl at a final concentration of 8 μM. In all the experiments, the EtOH concentration did not exceed 0.1%. The analog solutions were titrated with 2 μL (total 19 injections) of a 66 μM TgCyp23 solution supplemented with 0.1% EtOH. All the experiments were executed at an operating temperature of 20°C and a time gap of 150 s between each injection. Protein and ligand samples were filtered and degassed thoroughly before the experiments. Reference experiments were performed in the absence of a ligand or a protein, and the heat of dilution was subtracted from each experiment. Each titration set was repeated as a triplicate and the error was estimated as the SEM. The data were analyzed using the software MicroCal Origin, and the apparent dissociation constants K_d_, the enthalpy changes (ΔH), and entropy changes (ΔS) were obtained.

### 4.8 Circular dichroism spectroscopy and thermal denaturation profiles

Circular dichroism (CD) assays were conducted on a Jasco J‐1500 spectropolarimeter equipped with a Peltier temperature controller following the protocol described in (Bombardi et al., 2020; Favretto et al., 2023; Trande et al., 2019; ). Briefly, denaturation profiles in the presence of saturating concentrations of ligands were obtained by monitoring the loss of secondary structure of TgCyp23 at 224 nm in the temperature range of 15–100°C, with a scan rate of 1.5°C/min. The protein concentration was 0.2 mg/mL, and the buffer used was 50 mM HEPES at pH 8.0 with 100 mM NaCl.

### 4.9 Differential scanning calorimetry

Differential scanning calorimetry (DSC) experiments were conducted using a nano‐DSC (TA instrument) with a cell volume of 300 μL (Favretto et al., 2023). Protein samples, containing 70 μM TgCyp23 in 50 mM HEPES pH 8.0 and 100 mM NaCl in the presence or absence of CsA analogs, were heated from 15 to 100 °C at a scan rate of 1°C/min. Data were fitted according to a two‐state model using NanoAnalyze software (TA instrument).

### 4.10 PPIase inhibition studies

PPIase activity was determined using the synthetic peptide *N*‐succinyl‐Ala‐Ala‐Pro‐Phe‐*p*‐nitroanilide (AAPF) based on the protease‐coupled assay as described in (Favretto et al., 2023; Kofron et al., 1991). In inhibition assays, TgCyp23 (7 nM) was incubated for 10 min on ice in the presence of increasing concentrations of the three analogs, ranging from 0 to 30 nM, before the addition of the AAPF peptide. In all experiments, the slope of the catalyzed reaction was corrected for spontaneous thermal *cis–trans* isomerization in the presence of chymotrypsin alone.

The residual enzymatic activity was estimated from the initial velocities of each catalyzed reaction and expressed as a percentage (%) of the original enzymatic activity (Astegno et al., 2017). Residual enzymatic activities were plotted against ligand concentrations, and the obtained curve was analyzed to determine the IC_50_ value for each tested ligand. The experiments were repeated in triplicate, and the error was estimated as the standard error of the mean (SEM).

### 4.11 Stopped‐flow

Stopped‐flow experiments were performed on an SX‐18 stopped‐flow apparatus (Applied Photophysics, Leatherhead, UK) at 20°C in 50 mM Hepes, 150 mM NaCl, 1 mM DTT (pH 7.4). Fluorescence emission was measured using a 320 nm cutoff filter (λ_ex_ = 280 nm). The concentration of TgCyp23 after mixing was 0.5 μM, and the concentration of ligands after mixing ranged from 1 to 7 μM. For each trace, data from 4 to 6 injections were averaged and fitted with a single exponential function, excluding data points in the dead‐time of the stopped flow (<2 ms).

## AUTHOR CONTRIBUTIONS

**Filippo Favretto:** Conceptualization; investigation; funding acquisition; writing – review and editing; writing – original draft. **Eva Jiménez‐Faraco:** Investigation; writing – review and editing; writing – original draft; conceptualization. **Gianluca Catucci:** Investigation; writing – review and editing; funding acquisition. **Adele Di Matteo:** Investigation; writing – review and editing. **Carlo Travaglini‐Allocatelli:** Investigation; writing – review and editing. **Sheila J. Sadeghi:** Writing – review and editing; investigation. **Paola Dominici:** Writing – review and editing. **Juan A. Hermoso:** Conceptualization; funding acquisition; writing – original draft; writing – review and editing; supervision. **Alessandra Astegno:** Conceptualization; funding acquisition; writing – original draft; writing – review and editing; supervision.

## CONFLICT OF INTEREST STATEMENT

The authors declare no competing financial interest.

## Supporting information


**Table S1.** Binding energy and predicted binding affinity values from *in silico* docking of best‐scoring compounds.
**Table S2.** Data collection and refinement statistics for all TgCyp23 structures presented in this study.
**Table S3.** Polar contacts between TgCyp23 and CsA/dhCsA/NIM811/Alisporivir.
**Figure S1.** Interaction patterns of CsA analogs with TgCyp23.
**Figure S2.** 1H‐^15^N HSQC NMR spectra of ^15^N‐labeled TgCyp23 in the absence and presence of a twofold molar excess of CsA.
**Figure S3.** Chemical shift perturbation difference between TgCyp23:CsA complex and TgCyp23 in complex with the CsA analogs.
**Figure S4.** Interaction of TgCyp23 with CsA analogs studied by ITC.
**Figure S5.** Comparison of TgCyp23:CsA complex with *Homo sapiens* CypA:CsA complex (PDB: 1M63).
