# Supplementary material for: Evaluating the potential of non‐immunosuppressive cyclosporin analogs for targeting Toxoplasma gondii cyclophilin: Insights from structural studies
Source: Protein Sci. 2024 Sep 23;33(10):e5157. doi: 10.1002/pro.5157 (PMC11418636; doi:10.1002/pro.5157)
Supplement: Supplementary file 1 — Table S1. Binding energy and predicted binding affinity values from in silico docking of best‐scoring compounds. Table S2. Data collection and refinement statistics for all TgCyp23 structures presented in this study. Table S3. Polar contacts between TgCyp23 and CsA/dhCsA/NIM811/Alisporivir. Figure S1. Interaction patterns of CsA analogs with TgCyp23. Figure S2. 1H‐15N HSQC NMR spectra of 15N‐labeled TgCyp23 in the absence and presence of a twofold molar excess of CsA. Figure S3. Chemical shift perturbation difference between TgCyp23:CsA complex and TgCyp23 in complex with the CsA analogs. Figure S4. Interaction of TgCyp23 with CsA analogs studied by ITC. Figure S5. Comparison of TgCyp23:CsA complex with Homo sapiens CypA:CsA complex (PDB: 1M63). [file PRO-33-e5157-s001.pdf]

SUPPORTING INFORMATION

Evaluating the potential of non-immunosuppressive cyclosporin analogs for targeting *Toxoplasma gondii* cyclophilin: insights from structural studies

Filippo Favretto[1, #], Eva Jiménez-Faraco[2, #], Gianluca Catucci[3], Adele Di Matteo[4], Carlo Travaglini-Allocatelli[5], Sheila J. Sadeghi[3], Paola Dominici[1], Juan A. Hermoso[2, *], Alessandra Astegno[1, *]

[1]Department of Biotechnology, University of Verona, Strada Le Grazie 15, 37134 Verona, Italy.
[2] Department of Crystallography and Structural Biology, Institute of Physical Chemistry Blas Cabrera (IQF), CSIC, Serrano 119, 28006 Madrid, Spain.
[3]Department of Life Sciences and Systems Biology, University of Turin, 10123 Turin, Italy.
[4]CNR Institute of Molecular Biology and Pathology, P.le Aldo Moro 5, 00185 Rome, Italy.
[5]Department of Biochemical Sciences, Sapienza University of Rome, P.le Aldo Moro 5, 00185 Rome, Italy.

[#] These authors contributed equally.

*Corresponding authors. Alessandra Astegno: e-mail: alessandra.astegno@univr.it; Juan A. Hermoso: e-mail: xjuan@iqf.csic.es

Table of Contents

Table S1. Binding energy and predicted binding affinity values from *in silico* docking of best-scoring compounds.
Table S2. Data collection and refinement statistics for all TgCyp23 structures presented in this study.
Table S3. Polar contacts between TgCyp23 and CsA/dhCsA/NIM811/Alisporivir**.**
Figure S1. Interaction patterns of CsA analogs with TgCyp23.
Figure S2. ^{1}H-^{15}N HSQC NMR spectra of ^{15}N-labelled TgCyp23 in the absence and presence of a two-fold molar excess of CsA.
Figure S3. Chemical shift perturbation difference between the TgCyp23:CsA complex and TgCyp23 in complex with the CsA-analogs.
Figure S4. Interaction of TgCyp23 with CsA analogues studied by ITC.
Figure S5. Comparison of TgCyp23:CsA complex with *Homo sapiens* CypA:CsA complex (PDB: 1M63).

Table S1. Binding energy and predicted binding affinity values from *in silico* docking of best-scoring compounds.

	Compound name	Binding energy (kcal/mol)	Predicted Dissociation constant (nM)
1CWB[1]	*N*-Me-4-[(E)-2-butenyl]-4,4-dimethylthreonine-Cyclosporin A ($MeBm_2$ t1-CsA)	7.91	1586.45
1BCK[2]	Thr-2-Cyclosporin	7.69	2292.04
1CWA[3]	Cyclosporin A	7.68	2319.28
5TA4[4]	(Compound 8) based on Structural Simplification of Sanglifehrin A	7.56	2849.58
1CYN[5]	[d-(cholinylester)Ser8]-Cyclosporin	7.54	2967.38
1CWM[2]	MeIle-4-Cyclosporin (NIM811)	7.53	2987.48
5HSV[6]	MeAla-3-EtVal-4-Cyclosporin (Alisporivir)	7.47	3333.89
1CWL[2]	(4-hydroxy)-MeLeu-4-Cyclosporin	7.37	3907.09
Pubchem[7]	Dihydrocyclosporin A	7.34	4151.85
3ODL[8]	[(2s,3r,4r)-3-hydroxy-4-methyl-2-(methylamino)-6,8-nonadienoic Acid]-6-Cyclosporin A (Voclosporin Z-ISA247)	7.22	5024.26
1CWC[9]	Me[(4R,4S)-Me]norLeu-4-Cyclosporin	7.22	5049.77
1CWF[2]	Val-2-Cyclosporin	7.17	5503.70
5T9Z[4]	(Compound 6) based on Structural Simplification of Sanglifehrin A	7.1	6162.64
1NMK[10]	Sanglifehrin A	7.04	6830.94
1CWO[2]	Thr-2-Leu-5-Hiv-8-Leu-10-Cyclosporin	7.04	6865.61
1CWI[2]	Val-2-D-MeAla-3-Cyclosporin	6.93	8196.82
5TA2[4]	(Compound 7) based on Structural Simplification of Sanglifehrin A	6.81	10190.69
5T9W[4]	(Compound 5) based on Structural Simplification of Sanglifehrin A	6.77	10865.71
4TOT[11]	MeAla-3-(2-methoxyethyl)piperazine-4-Cyclosporin (NIM258)	6.61	14090.99
1CWJ[2]	*Val-2-*D-(2-*S-Me*)*Sar-3-Cyclosporin*	6.6	14330.84
1CWK[2]	(6,7-dihydro)MeBmt-1-Val-2-D-(2-S-Me)Sar-3-Cyclosporin	6.5	17167.37
5A0E[12]	[Gly-(1S,2R,E)-8-quinolinium-1-hydroxy-2-methyloct-4-ene]-1-Cyclosporin (JW47)	6.33	22834.02
1CWH[2]	MeSer-3-Cyclosporin	5.4	109718.91

Table S2. Data collection and refinement statistics for all TgCyp23 structures presented in this study.

Data collection [a]	**TgCyp23:NIM811**	**TgCyp23:dhCsA**	**TgCyp23:Ali**
Space group	$P2_1$	$P2_1$	$P2_1$
Cell dimensions			
a, b, c (Å)	*a*=38.09, *b*=118.09, *c*=45.88	*a*=38.35, *b*=118.11, *c*=47.46	*a*=38.31, *b*=118.52, *c*=47.53
α, β, γ (°)	90, 103.27, 90	90, 103.67, 90	90, 103.52, 90
Wavelength (Å)	0.97926	0.97926	0.97926
Resolution (Å)	44.66-1.17 (1.21-1.17)	42.96 - 1.20 (1.24 - 1.20)	42.27 -1.20 (1.24-1.20)
unique reflections	131489 (12900)	125896 (12366)	125282 (12479)
[b] R_{merge}	0.064 (0.761)	0.046 (1.028)	0.054 (1.000)
[c] R_{pim}	0.027 (0.331)	0.019 (0.418)	0.023 (0.432)
$CC_{1/2}$	0.999 (0.807)	0.999 (0.752)	0.999 (0.701)
mean $I/\sigma(I)$	14.3 (2.2)	16.8 (1.6)	15.0 (1.6)
Completeness (%)	99.2 (97.8)	98.7 (97.0)	99.9 (99.9)
Multiplicity	6.6 (6.1)	6.8 (6.8)	6.2 (6.2)
Refinement			
Resolution (Å)	44.66-1.17	42.96-1.20	42.27-1.20
[d] R_{work}/R_{free}	0.148/0.168	0.172/0.187	0.164/0.179
no. Of atoms			
Total	3846	3721	3924
Protein	3141	3103	3133
Ligands	170	170	172
solvent	535	448	619
Ramachandran Analysis			
Ramachandran favored (%)	98.48	98.48	98.21
Ramachandran outliers (%)	0.00	0.00	0.00
average B, all atoms ($Å^2$)	18.1	22.0	18.0
Root-mean-square deviation (RMSD)			
bond lengths (Å)	0.010	0.006	0.006
bond angles (°)	1.16	0.92	0.94
PDB entry	**8R7S**	**8R7U**	**8R7T**

[a]Values between parentheses correspond to the highest resolution shells.
[b]**R**merge _ _hkl _i | Ii(hkl) – [I(hkl)] |/_hkl_iIi(hkl), where _i**I**i(hkl) is the **i**-th measurement of reflection hkl and [I(hkl)] is the weighted mean of all measurements.
[c]**R**pim _ _hkl[1/(**N** – 1)]1/2 _i | **I**i(hkl) – [**I**(hkl)] |/_hkl_i**I**i(hkl), where _i**I**i(hkl) is the **i**-th measurement of reflection hkl, [**I**(hkl)] is the weighted mean of all measurements, and **N** is the redundancy for the hkl reflection.
[d]**R**work/**R**free _ _hkl| **F**o – **F**c |/_hkl | **F**o |, where **F**c is the calculated and **F**o is the observed structure factor amplitude of reflection hkl for the working/free (5%) set, respectively.

Table S3. Polar contacts between TgCyp23 and CsA/dhCsA/NIM811/Alisporivir

TgCyp23 residues/ atoms (Chain B)		CsA residues/atoms (Chain D)		Distance (Å)
Arg99	NH1	MeLeu-10	O	2.9
Arg99	NH2	MeLeu-10	O	2.9
Gln107	NE-2	MeBmt-1	O	3.1
Gly116	O	Sar-3	N	3.1
Ser147	OG	Abu-2	O	2.9
Asn146	O	Abu-2	N	3.0
Asn146	N	MeVal-11	O	3.5
Trp165	NE1	MeLeu-9	O	2.9
His170	NE2	MeVal-11	O	3.4
**TgCyp23 residues/ atoms (Chain B)**		**dhCsA residues/atoms (Chain B)**		**Distance (Å)**
Arg99	NH1	MeLeu-10	O	2.9
Arg99	NH2	MeLeu-10	O	2.8
Gln107	NE-2	MeBmt-1	O	3.0
Gly116	O	Sar-3	N	3.0
Ser147	OG	Abu-2	O	2.9
Asn146	O	Abu-2	N	3.0
Asn146	N	MeVal-11	O	3.4
Trp165	NE1	MeLeu-9	O	2.7
His170	NE2	MeVal-11	O	3.3
**TgCyp23 residues/ atoms (Chain B)**		**NIM811 residues/atoms (Chain B)**		**Distance (Å)**
Arg99	NH1	MeLeu-10	O	2.9
Arg99	NH2	MeLeu-10	O	2.9
Gln107	NE-2	MeBmt-1	O	3.0
Gly116	O	Sar-3	N	3.1
Ser147	OG	Abu-2	O	2.7
Asn146	O	Abu-2	N	3.0
Asn146	N	MeVal-11	O	3.4
Trp165	NE1	MeLeu-9	O	2.8
His170	NE2	MeVal-11	O	3.4
**TgCyp23 residues/ atoms (Chain B)**		**Ali residues/atoms (Chain B)**		**Distance (Å)**
Arg99	NH1	MeLeu-10	O	3.0
Arg99	NH2	MeLeu-10	O	2.9
Gln107	NE-2	MeBmt-1	O	3.0
Gly116	O	Sar-3	N	3.2
Ser147	OG	Abu-2	O	2.8
Asn146	O	Abu-2	N	3.2
Asn146	N	MeVal-11	O	3.3
Trp165	NE1	MeLeu-9	O	2.8
His170	NE2	MeVal-11	O	3.4

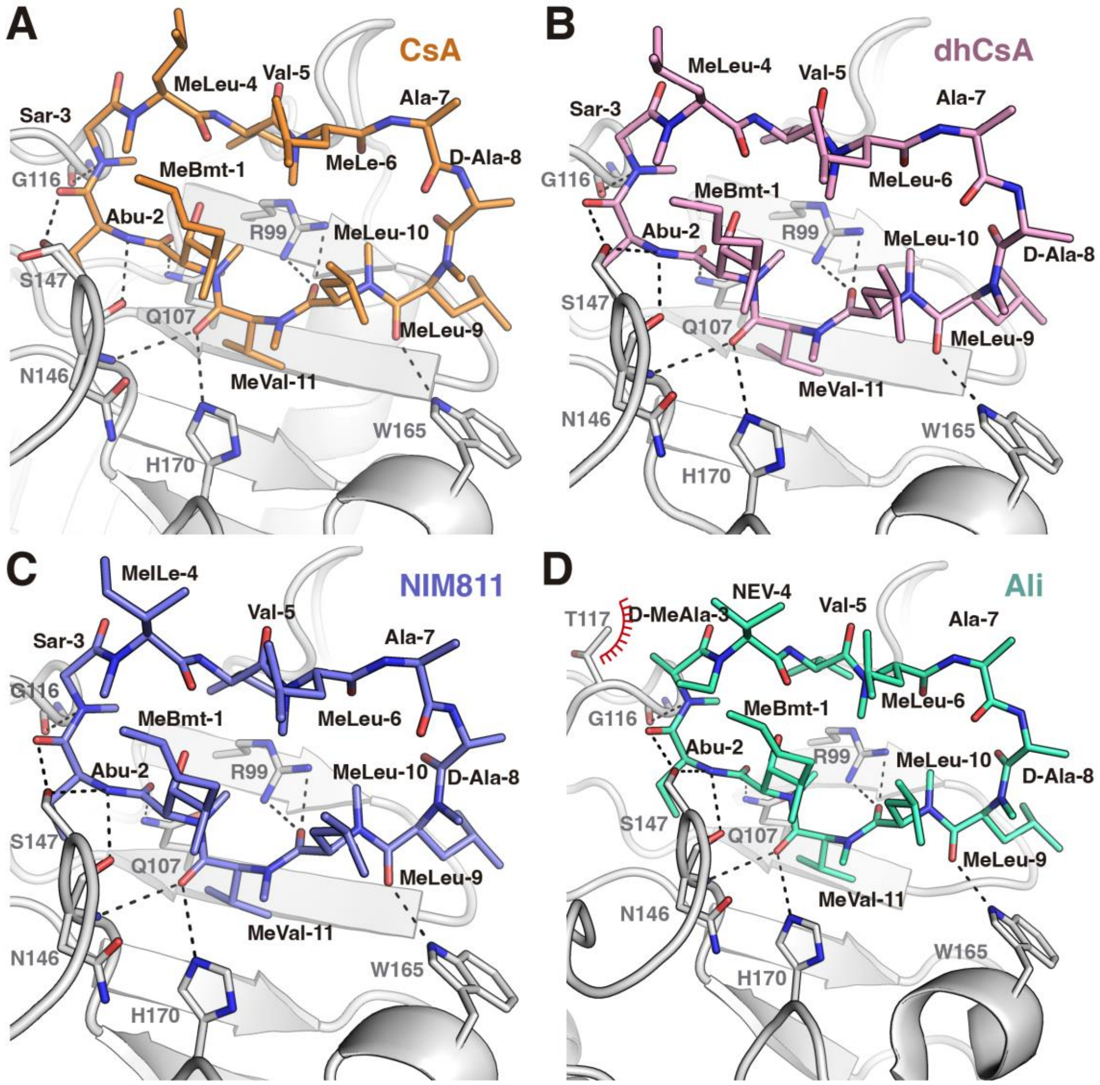

Figure S1. **Interaction patterns of CsA analogs with TgCyp23.** (**A)** TgCyp23:CsA complex, CsA in orange sticks. (**B)** TgCyp23:dhCsA complex, dhCsA in pink sticks. (**C)** TgCyp23:NIM811 complex, NIM811 in purple sticks. (**D)** TgCyp23:Alisporivir complex, Ali in cyan sticks. The protein is displayed as a grey cartoon, and relevant residues implicated in the interaction are labeled and shown as sticks. Black dashed lines indicate polar interactions, and the red dashed line represents Van der Waals forces.

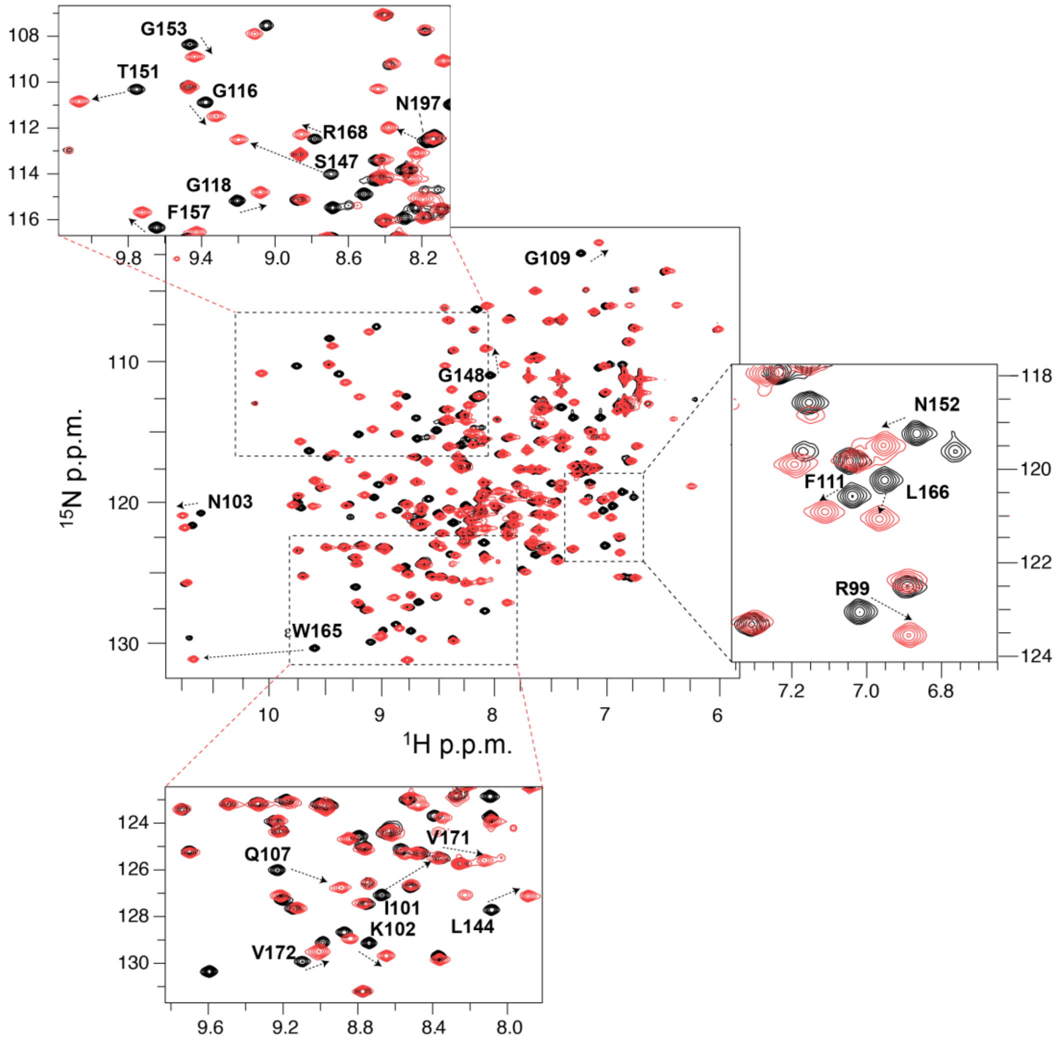

Figure S2. ^{1}H-^{15}N HSQC NMR spectra of ^{15}N-labelled TgCyp23 in the absence (black) and presence of a two-fold molar excess of CsA (red). Enlarged views of selected spectral regions that show significant chemical shift perturbation upon CsA binding are displayed. Significantly perturbed protein residues are also labelled with single-letter amino acid code.

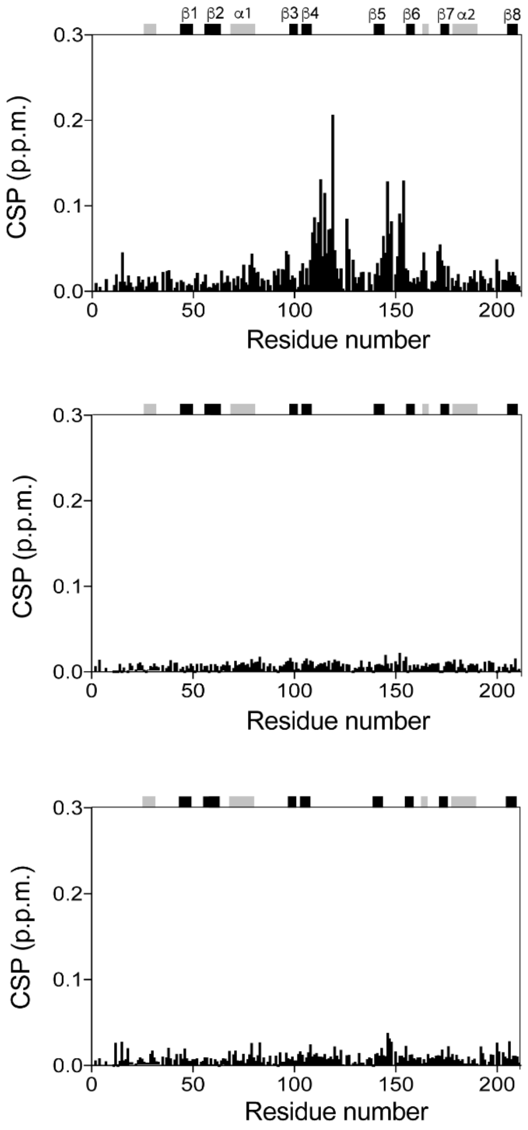

Figure S3. Chemical shift perturbation difference between the TgCyp23:CsA complex and TgCyp23 in complex with the CsA-analogs. Alisporivir (upper panel), NIM811 (middle panel) and dhCsA (lower panel). Secondary structure elements are displayed.

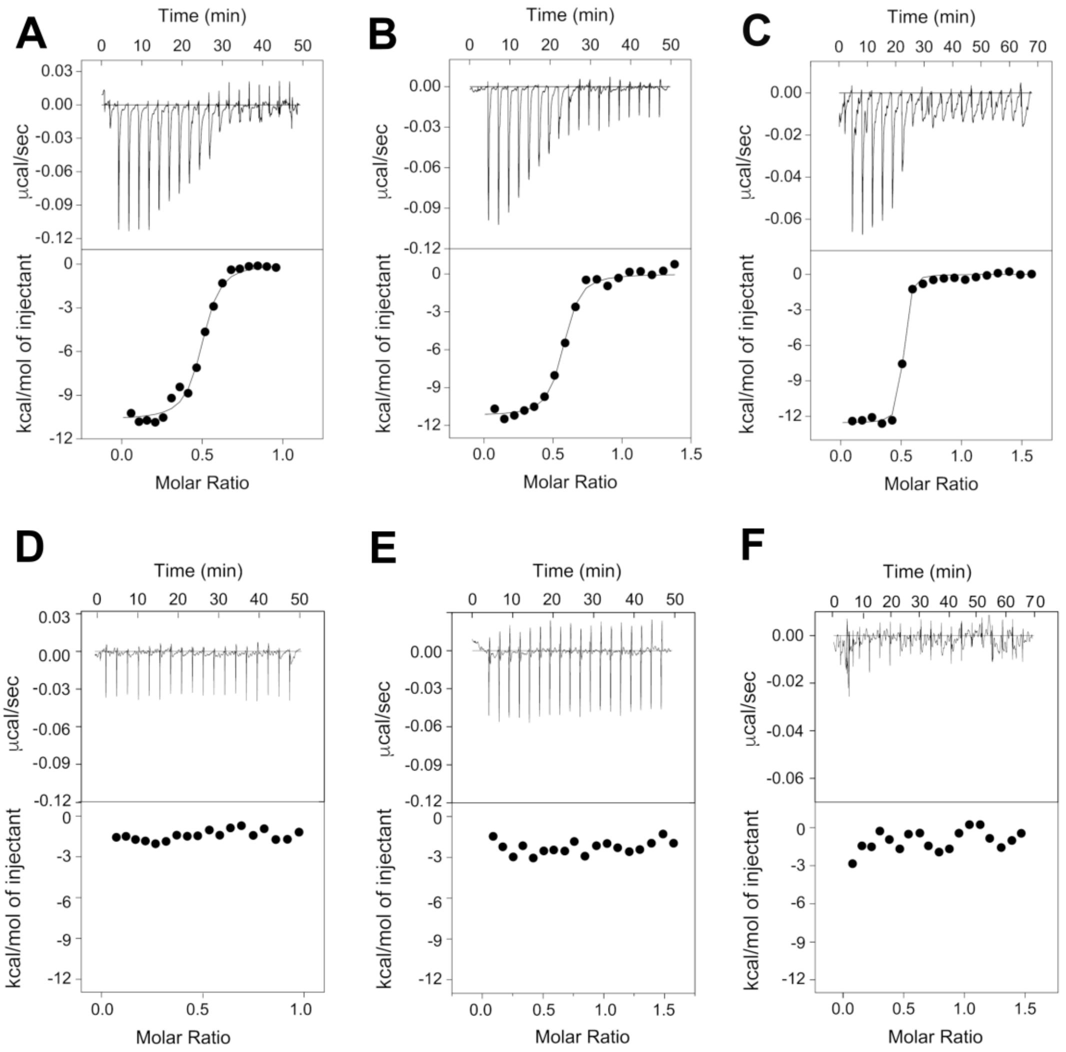

Figure S4. Interaction of TgCyp23 with CsA analogs studied by ITC. (A-C) Representative thermograms (top panels) and the derived binding isotherms (bottom panels) of TgCyp23 in complex with dhCsA (**A**), NIM811 (**B**) and Alisporivir (**C**) recorded at 20 °C. (**D-E**) Blank titrations were performed by injecting dhCsA (**D**), NIM811 (**E**), and Alisporivir (**F**) into the buffer devoid of protein to correct for the heat of mixing and ligand dilution. This contribution to the observed heat of reaction was subtracted from the corresponding total heat.

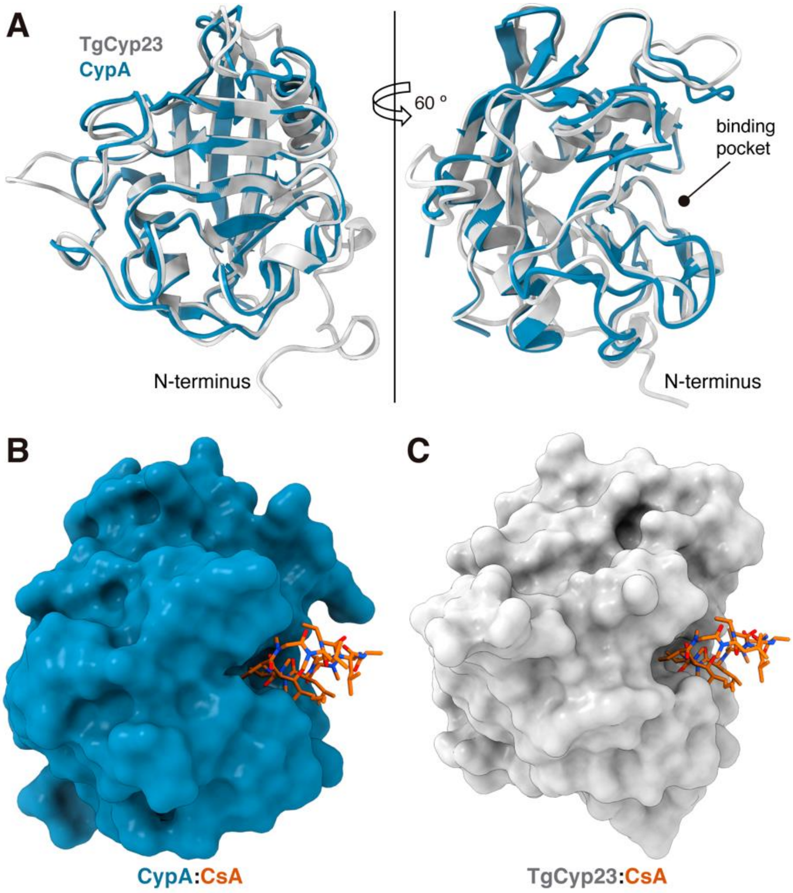

Figure S5. Comparison of TgCyp23:CsA complex with *Homo sapiens* CypA:CsA complex (PDB: 1M63). (**A)** Alignment between CypA (Blue) and TgCyp23 (grey). Structures are displayed in cartoon view. Right panel shows a 60º rotated view. (**B)** Representation of the molecular surface of CypA (colored in blue) showing the CsA binding pocket (CsA depicted as orange capped sticks). (**C)** Representation of the molecular surface of TgCyp23 (colored in grey) showing the CsA binding pocket (CsA depicted as orange capped sticks).

References

1. Mikol, V.; Kallen, J.; Walkinshaw, M. D., The X-ray structure of (MeBm2t)1-cyclosporin complexed with cyclophilin A provides an explanation for its anomalously high immunosuppressive activity. *Protein engineering* **1994,** *7* (5), 597-603.
2. Kallen, J.; Mikol, V.; Taylor, P.; Walkinshaw, M. D., X-ray structures and analysis of 11 cyclosporin derivatives complexed with cyclophilin A. *Journal of molecular biology* **1998,** *283* (2), 435-49.
3. Mikol, V.; Kallen, J.; Pflügl, G.; Walkinshaw, M. D., X-ray structure of a monomeric cyclophilin A-cyclosporin A crystal complex at 2.1 A resolution. *Journal of molecular biology* **1993,** *234* (4), 1119-30.
4. Steadman, V. A.; Pettit, S. B.; Poullennec, K. G.; Lazarides, L.; Keats, A. J.; Dean, D. K.; Stanway, S. J.; Austin, C. A.; Sanvoisin, J. A.; Watt, G. M.; Fliri, H. G.; Liclican, A. C.; Jin, D.; Wong, M. H.; Leavitt, S. A.; Lee, Y.-J.; Tian, Y.; Frey, C. R.; Appleby, T. C.; Schmitz, U.; Jansa, P.; Mackman, R. L.; Schultz, B. E., Discovery of Potent Cyclophilin Inhibitors Based on the Structural Simplification of Sanglifehrin A. *Journal of medicinal chemistry* **2017,** *60* (3), 1000-1017.
5. Mikol, V.; Kallen, J.; Walkinshaw, M. D., X-ray structure of a cyclophilin B/cyclosporin complex: comparison with cyclophilin A and delineation of its calcineurin-binding domain. *Proceedings of the National Academy of Sciences of the United States of America* **1994,** *91* (11), 5183-6.
6. Dujardin, M.; Bouckaert, J.; Rucktooa, P.; Hanoulle, X., X-ray structure of alisporivir in complex with cyclophilin A at 1.5 Å resolution. *Acta crystallographica. Section F, Structural biology communications* **2018,** *74* (Pt 9), 583-592.
7. Kim, S.; Chen, J.; Cheng, T.; Gindulyte, A.; He, J.; He, S.; Li, Q.; Shoemaker, B. A.; Thiessen, P. A.; Yu, B.; Zaslavsky, L.; Zhang, J.; Bolton, E. E., PubChem 2023 update. *Nucleic Acids Research* **2022,** *51* (D1), D1373-D1380.
8. Kuglstatter, A.; Mueller, F.; Kusznir, E.; Gsell, B.; Stihle, M.; Thoma, R.; Benz, J.; Aspeslet, L.; Freitag, D.; Hennig, M., Structural basis for the cyclophilin A binding affinity and immunosuppressive potency of E-ISA247 (voclosporin). *Acta crystallographica. Section D, Biological crystallography* **2011,** *67* (Pt 2), 119-23.
9. Papageorgiou, C.; Florineth, A.; Mikol, V., Improved binding affinity for cyclophilin A by a cyclosporin derivative singly modified at its effector domain. *Journal of medicinal chemistry* **1994,** *37* (22), 3674-6.
10. Sedrani, R.; Kallen, J.; Martin Cabrejas, L. M.; Papageorgiou, C. D.; Senia, F.; Rohrbach, S.; Wagner, D.; Thai, B.; Jutzi Eme, A.-M.; France, J.; Oberer, L.; Rihs, G.; Zenke, G.; Wagner, J., Sanglifehrin−Cyclophilin Interaction: Degradation Work, Synthetic Macrocyclic Analogues, X-ray Crystal Structure, and Binding Data. *Journal of the American Chemical Society* **2003,** *125* (13), 3849-3859.
11. Fu, J.; Tjandra, M.; Becker, C.; Bednarczyk, D.; Capparelli, M.; Elling, R.; Hanna, I.; Fujimoto, R.; Furegati, M.; Karur, S.; Kasprzyk, T.; Knapp, M.; Leung, K.; Li, X.; Lu, P.; Mergo, W.; Miault, C.; Ng, S.; Parker, D.; Peng, Y.; Roggo, S.; Rivkin, A.; Simmons, R. L.; Wang, M.; Wiedmann, B.; Weiss, A. H.; Xiao, L.; Xie, L.; Xu, W.; Yifru, A.; Yang, S.; Zhou, B.; Sweeney, Z. K., Potent nonimmunosuppressive cyclophilin inhibitors with improved pharmaceutical properties and decreased transporter inhibition. *Journal of medicinal chemistry* **2014,** *57* (20), 8503-16.
12. Warne, J.; Pryce, G.; Hill, J. M.; Shi, X.; Lennerås, F.; Puentes, F.; Kip, M.; Hilditch, L.; Walker, P.; Simone, M. I.; Chan, A. W.; Towers, G. J.; Coker, A. R.; Duchen, M. R.; Szabadkai, G.; Baker, D.; Selwood, D. L., Selective Inhibition of the Mitochondrial Permeability Transition Pore Protects against Neurodegeneration in Experimental Multiple Sclerosis. *The Journal of biological chemistry* **2016,** *291* (9), 4356-73.
